# ERK2 Suppresses Self-Renewal Capacity of Embryonic Stem Cells, but Is Not Required for Multi-Lineage Commitment

**DOI:** 10.1371/journal.pone.0060907

**Published:** 2013-04-16

**Authors:** William B. Hamilton, Keisuke Kaji, Tilo Kunath

**Affiliations:** MRC Centre for Regenerative Medicine, University of Edinburgh, Edinburgh, United Kingdom; National University of Singapore, Singapore

## Abstract

Activation of the FGF-ERK pathway is necessary for naïve mouse embryonic stem (ES) cells to exit self-renewal and commit to early differentiated lineages. Here we show that genetic ablation of *Erk2*, the predominant ERK isozyme expressed in ES cells, results in hyper-phosphorylation of ERK1, but an overall decrease in total ERK activity as judged by substrate phosphorylation and immediate-early gene (IEG) induction. Normal induction of this subset of canonical ERK targets, as well as p90RSK phosphorylation, was rescued by transgenic expression of either ERK1 or ERK2 indicating a degree of functional redundancy. In contrast to previously published work, *Erk2*-null ES cells exhibited no detectable defect in lineage specification to any of the three germ layers when induced to differentiate in either embryoid bodies or in defined neural induction conditions. However, under self-renewing conditions *Erk2*-null ES cells express increased levels of the pluripotency-associated transcripts, *Nanog* and *Tbx3*, a decrease in *Nanog*-GFP heterogeneity, and exhibit enhanced self-renewal in colony forming assays. Transgenic add-back of ERK2 is capable of restoring normal pluripotent gene expression and self-renewal capacity. We show that ERK2 contributes to the destabilization of ES cell self-renewal by reducing expression of pluripotency genes, such as *Nanog*, but is not specifically required for the early stages of germ layer specification.

## Introduction

Pluripotent mouse embryonic stem (ES) cells are immortal, karyotypically stable, cell lines derived from the inner cell mass (ICM) or early epiblast of pre-implantation embryos [1]–[3]. They maintain many of the characteristics of their *in vivo* counterparts even after prolonged periods of culture, not least of which is the ability to generate differentiated cell types of all three germ layers as well as contribute to the germ line [4]. The efficient derivation and maintenance of ES cells is heavily dependent on genetic background [5], [6], and supported by the combined activities of leukemia inhibitory factor (LIF) and bone morphogenetic protein (BMP) signaling [7]. Both genetic studies [8], [9] and whole transcriptome analysis [10] have provided a wealth of information as to the factors that maintain ES cells in an undifferentiated state, and the signaling networks that regulate their expression [11]. Our current understanding is that the combined activities of a core network consisting of the transcription factors OCT4, SOX2 and Nanog act to maintain ES cell identity by inhibiting the expression of lineage affiliated genes [12] and supporting a pluripotent epigenetic signature [10].

Although much is now known about the molecular requirements for efficient ES cell self-renewal, the means by which cells exit this state and acquire the competence to respond to differentiation cues is less well understood. Genetic studies have shown that fibroblast growth factor (FGF) signaling through GRB2-RAS is essential for peri-implantation mouse embryogenesis and the formation of the primitive endoderm lineage [13]–[16]. The ES cell, and presumably ICM/epiblast expression of *Fgf4* is driven by a ternary complex consisting of OCT4, SOX2 and the *Fgf4* distal enhancer [17], and acts as a major auto-inductive cue for ES cell differentiation, but is largely dispensable for proliferation [18], [19]. Moreover, it has been shown that the combined pharmacological inhibition of FGF-ERK and glycogen synthase kinase 3 (GSK3) signaling pathways promotes long-term self-renewal of ES cells in the absence of both LIF and BMP4 [7]. This dual inhibitor (2i) culture condition permitted the derivation of ES cell lines from previously refractory genetic backgrounds such as CBA [5], and non-obese diabetic (NOD) mice [20], and importantly the establishment of authentic rat ES cells [21], [22].

Because ERK2 is the predominant ERK enzyme expressed in ES cells, and because of its early embryonic phenotype [23], [24], we sought to determine the effect of *Erk2* genetic ablation on FGF signaling, self-renewal and lineage specification. Here we present evidence that ERK2 is necessary for maximal FGF-ERK signaling and its deletion results in a modest, but consistent increase in self-renewal capacity and reduction in heterogeneity of *Nanog* expression in self-renewing culture conditions. In contrast to our previous work which indicated a requirement for ERK2 in both neural and mesodermal specification [18], we found that our new *Erk2^−/−^* ES cells did not exhibit any significant defects in germ layer specification when induced to differentiate. Either ERK1 or ERK2 could rescue the FGF signaling defects in *Erk2^−/−^* cells suggesting these enzymes share some functional redundancy in mouse ES cells. We propose that total combined ERK activity impacts on ES cell self-renewal by reducing pluripotent gene expression and that ERK1 and ERK2 share overlapping functions during the earliest stages of ES cell differentiation.

## Results

### Targeting *Erk2* in Mouse ES Cells

**Figure 1:**
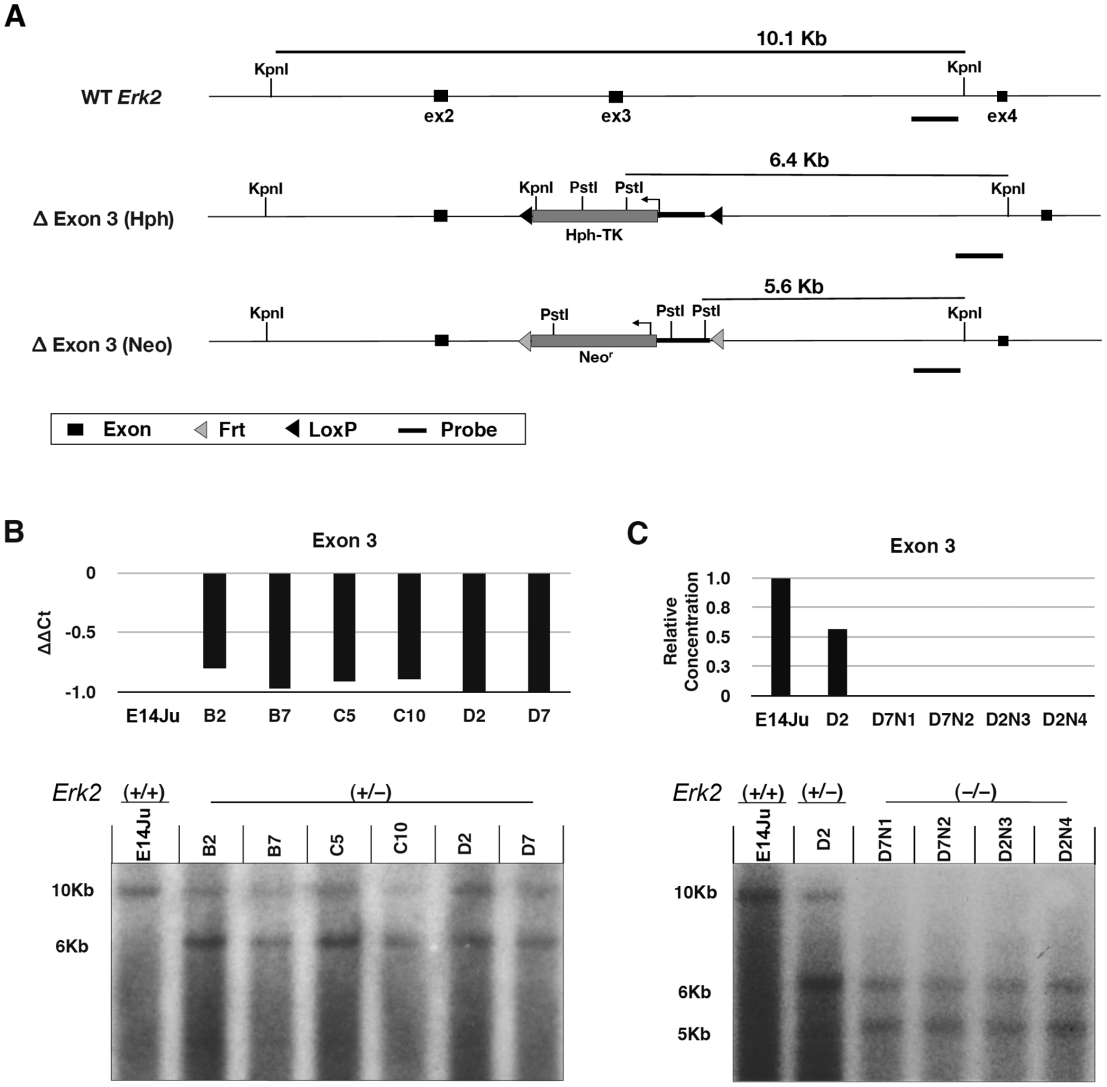
*Erk2* gene targeting. (A) Schematic illustrating the WT *Erk2* locus, and both Δexon 3 (Hph and Neo) targeted alleles. Black bars indicate the location of the 3' probe used for Southern blotting. Sizes of the KpnI and KpnI-PstI fragments for the WT and targeted alleles, respectively, are shown. *Hph-TK*: hygromycin B phosphotransferase-thymidine kinase fusion gene; *Neo^r^*: neomycin resistance gene. (B) Correct targeting in heterozygous ES cells was screened by genomic qPCR for loss of a single exon 3 allele (top), and confirmed by Southern blotting (bottom). (C) The second targeting to generate homozygous *Erk2*-null ES cells was also screened by genomic qPCR (top), and confirmed by Southern blotting (bottom).

To disrupt *Erk2* function, germ-line competent 129/Ola ES cells (E14Ju) were depleted for exon 3 of the *Erk2* gene by two round of homologous recombination (Figure 1A). This strategy removes the region coding for kinase sub-domains V and VI, essential for ERK2 function, and has been previously shown to create a protein-null *Erk2* allele [23], [25]. Heterozygous ES cell clones were first screened for reduction of exon 3 alleles by quantitative PCR (qPCR) and putative targeted lines were confirmed by Southern blotting (Figure 1B). Two heterozygous *Erk2* ^+/−^ ES cell clones (D2 and D7) were subjected to a second round of homologous recombination and clones were first screened for complete loss of exon 3 by qPCR, and further confirmed by Southern blotting (Figure 1C). All clones were karyotyped following clonal expansion and retained normal ploidy (Figure S1). Two *Erk2*-null clones, D7N2 and D2N4, each derived from independent heterozygous clones, D7 and D2, respectively, were selected for further analysis.

**Figure 2:**
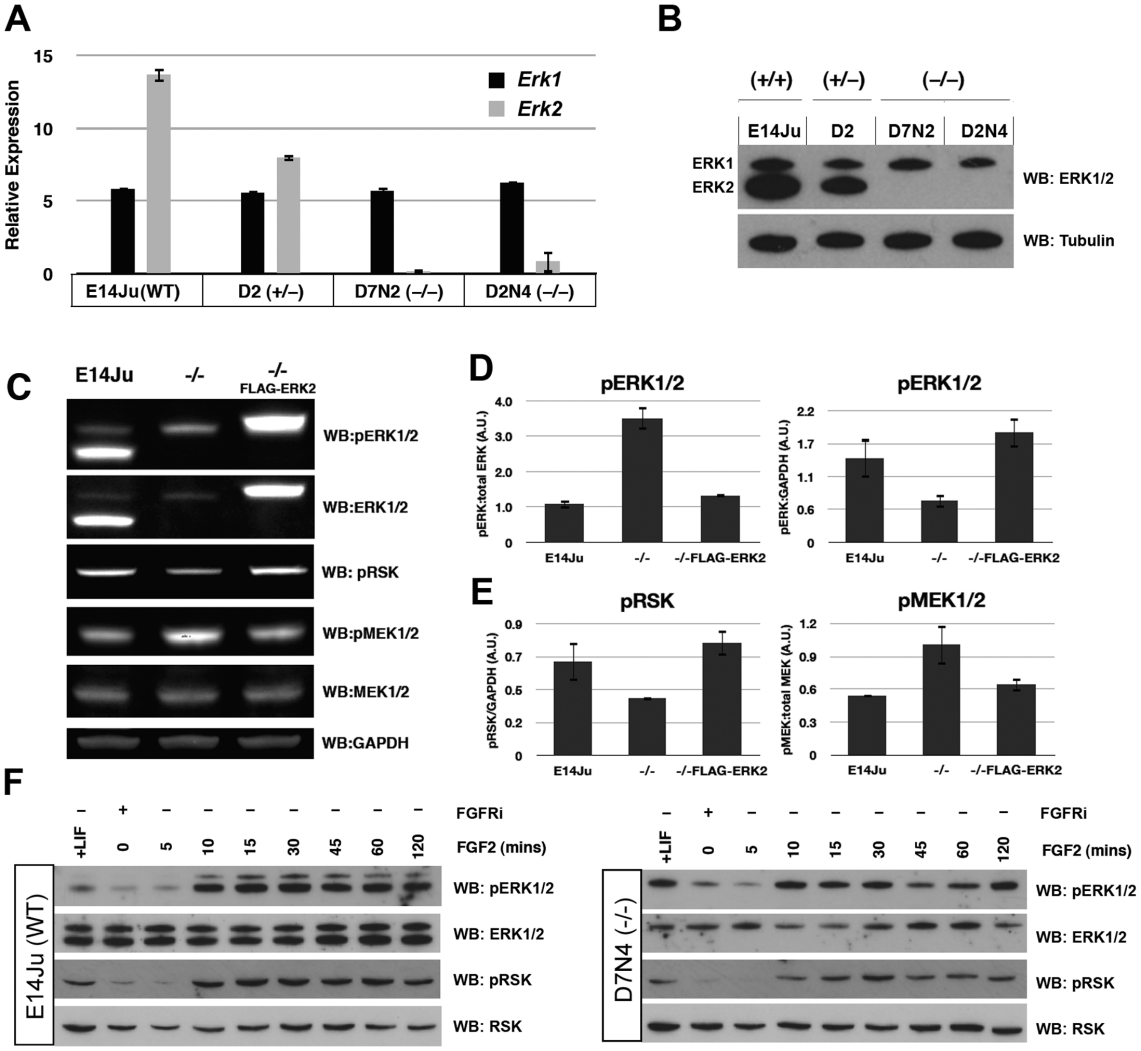
ERK2 ablation attenuates FGF-ERK signaling. (A) qRT-PCR analysis showing reduced or absent *Erk2* transcript in the mutated lines, with no compensatory increase in *Erk1* expression. (B) Western blot analysis of total ERK1/2 protein levels in WT, heterozygous, and homozygous *Erk2* cell lines. ERK2 expression is reduced in the heterozygous line D2, and completely absent in the *Erk2* ^−/−^ lines D7N2 and D2N4. Tubulin is used as a loading control. The qRT-PCR and western results for the heterozygous line D7 were identical to clone D2 (not shown). (C) Western blot analysis of wild-type (E14Ju), *Erk2* ^−/−^, FLAG-ERK2-rescued *Erk2* ^−/−^ ES cells. The levels of phospho-ERK1/2, phospho-RSK and phospho-MEK1/2 were examined in self-renewing culture conditions. (D) Quantification of western blotting data to examine the ratio of phosphorylated ERK1/2 to total ERK1/2 (left), and pERK1/2 to GAPDH (right) in E14Ju, *Erk2* ^−/−^, and ERK2-rescued *Erk2* ^−/−^ lines. (E) Quantification of western blotting data to examine the ratio of phosphor-RSK to GAPDH (left), and phospo-MEK1/2 to total MEK1/2 (right) in E1Ju, *Erk2* ^−/−^, and ERK2-rescued *Erk2* ^−/−^ lines. (F) Western blot analysis of ERK1/2 and RSK activation in wild-type (E14Ju) and *Erk2* ^−/−^ ES cells following acute FGF2 stimulation. Phosphorylation of p90RSK is reduced in the absence of ERK2. Membranes were stripped and sequentially blotted with the indicated antibodies.

To test whether the targeted mutation generated the predicted null allele, *Erk2* expression was assessed by qRT-PCR and ERK2 protein by western blotting. A 50% reduction of *Erk2* expression was observed in heterozygous *Erk2* ^+/−^ ES cells, and an absence of mRNA from both homozygous null lines (Figure 2A). Moreover, there was no evidence of a compensatory increase in *Erk1* mRNA expression. This was also confirmed on the protein level by immunoblotting with a polyclonal antibody that recognizes both ERK1 and ERK2 (Figure 2B).

### ERK2 Depletion Attenuates Short Term, but not Long Term FGF-ERK Signaling

To test whether ERK signaling was perturbed in the absence of ERK2, lysates from WT and *Erk2^−/−^* ES cells, as well as *Erk2^−/−^* cells expressing FLAG-ERK2 from a randomly integrated transgene were examined for phosphorylation status of the ERK, MEK, and p90RSK enzymes. In steady-state self-renewing conditions ERK2 depletion resulted in a 3-fold increase in the ratio of phospho-ERK to total ERK, which was returned to a normal ratio in FLAG-ERK2 expressing cells (Figure 2C,D). However, the amount of total phospho-ERK, i.e. pERK1(+pERK2)/GAPDH, was decreased by approximately 50% in ERK2-deficient ES cells (Figure 2D). Moreover the decrease in total phospho-ERK in *Erk2* knock-out cells translated into a similar decrease in phospho-p90RSK levels on the ERK-dependent sites T358 and S362 (Figure 2E) [26]. Interestingly, depletion of ERK2 resulted in an increase in the levels of phospho-MEK1/2 (upstream ERK kinases) at S211 and S217 (Figure 2E), suggesting that the observed increase in ERK1 phosphorylation in *Erk2*-null ES cells was due to decreased ERK-dependent negative feedback up-stream in the kinase cascade [27]. To examine FGF responsiveness in the absence of ERK2 we inhibited endogenous FGF signaling with a chemical inhibitor (PD173074) for 24 h followed by a wash-out and stimulation with recombinant FGF2 for 5–120 min. This acute stimulation with FGF2 exhibited similar time-to-peak (10–30 min) and decay kinetics in the presence or absence of ERK2 (Figure 2F). However, the ERK2-deficient ES cells exhibited a decrease in p90RSK phosphorylation indicating a reduction in the overall output of ERK signaling (Figure 2F).

**Figure 3:**
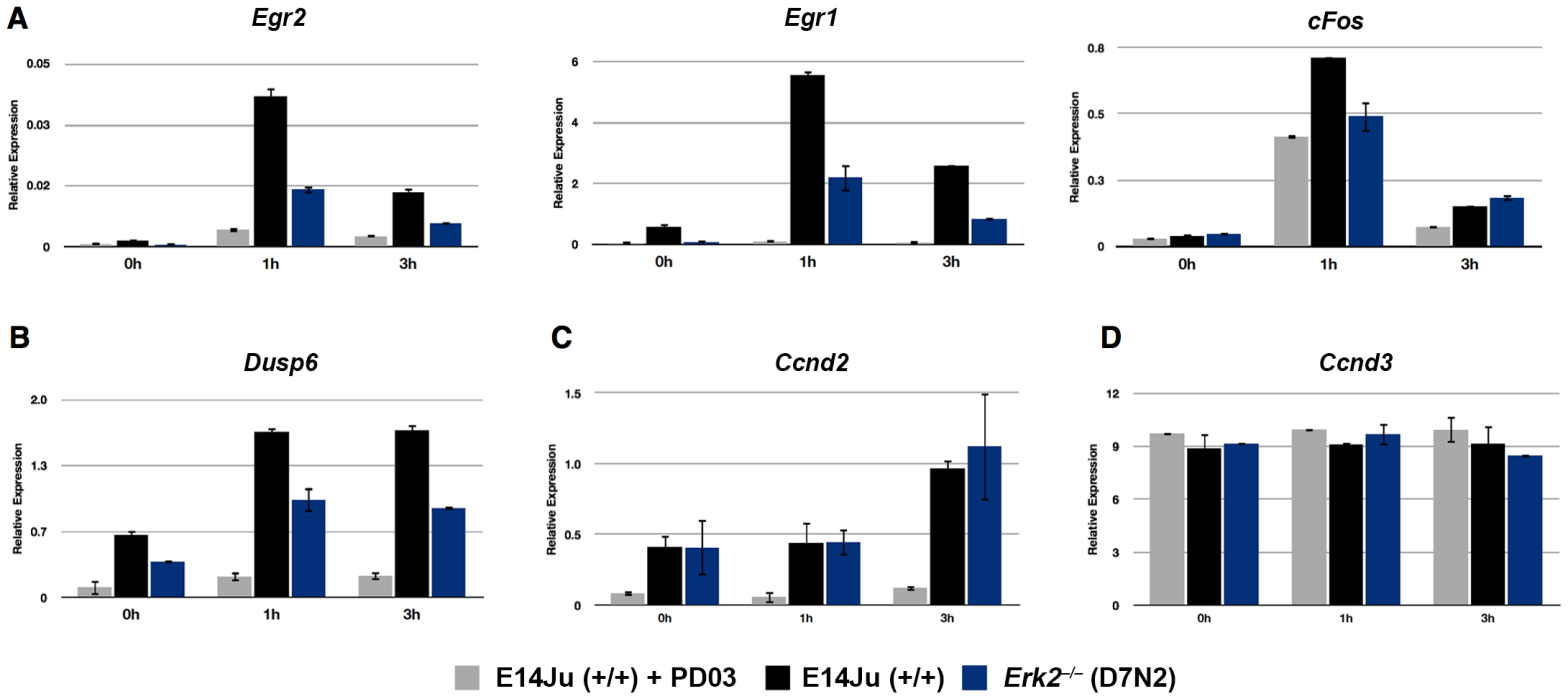
Attenuation of immediate early gene (IEG) induction in ERK2-deficient ES cells. (A) Analysis of IEG induction by qRT-PCR for *Egr2*, *Egr1*, and *cFos* following acute stimulation with FGF2. WT (E14Ju) and *Erk2*-null (D7N2) ES cells were cultured for 24 h in the presence of the FGFR inhibitor PD173074 (PD17). The inhibitor was then washed out (0 h), and cells were stimulated with (10 ng/ml) recombinant FGF2 for 1 and 3 hours. To determine basal MEK-ERK independent expression of the genes, WT cells were stimulated with media containing both FGF2 and the MEK1 inhibitor PD0325901 (PD03) (E14Ju+PD03) (B–D) FGF2 induction results for the ERK1/2 phosphatase *Dusp6* (B), the late response gene *Ccnd2* (C), and *Ccnd3* (D). Error bars represent the standard deviation from the mean of biological replicates.

As both ERK1/2 and p90RSK activities have been shown to be essential for the induction of immediate-early genes (IEGs) *cFos*, *Egr1*, and *Egr2* in other systems [28], [29], we next asked if this global reduction in ERK activity was reflected in the transcriptional response to FGF stimulation. qRT-PCR analysis of IEGs *Egr1* and *Egr2* expression following acute stimulation with FGF2 showed an approximately 70% reduction in their induction at 1 hour post-stimulation in ERK2-deficient ES cells consistent with a reduction in FGF pathway activity (Figure 3A). The induction of another IEG, *cFos*, was only reduced by approximately 35% in *Erk2* ^−/−^ ES cells (Figure 3A). However, its induction was similar to FGF stimulation of WT cells in the presence of the MEK inhibitor PD0325901. This indicated that only a fraction of *cFos* induction was MEK-dependent, with the MEK-independent *cFos* expression possibly being due to FGF-mediated phosphatidylinositol 3′-OH kinase (PI3K)-AKT induction of *cFos* [30].

*Dusp6* expression and its protein stability are both regulated by ERK activity [31], [32] where they act in a negative feedback loop that regulates the duration of ERK activity [29]. As the duration of ERK activity can determine the expression of late response genes [33], we asked if the expression of *Dusp6* was attenuated in the absence of ERK2 and if this correlated with a difference in late response gene expression such as D-type cyclins. Similar to *Egr1, Egr2,* and *cFos*, the magnitude of *Dusp6* expression was reduced in *Erk2* ^−/−^ ES cells (Figure 3B), however FGF2-induced, MEK-dependent, *Ccnd2* expression was not affected by loss of ERK2 (Figure 3C) indicating that the initial decrease in pathway activity can be compensated for in the long term, possibly by a decreased amount of induced negative feedback. Consistent with studies into the unique cell cycle architecture of mouse ES cells [34], *Ccnd3* expression was independent of both ERK and MEK activity (Figure 3D).

**Figure 4:**
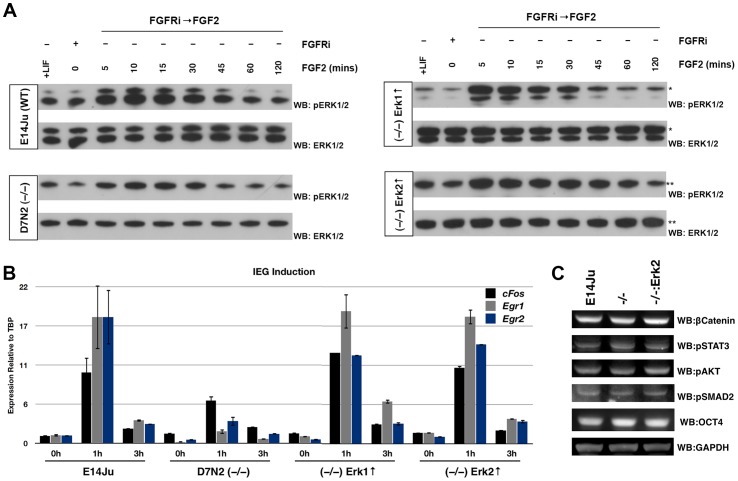
Overexpression of either ERK isozyme is capable of rescuing the IEG induction phenotype of *Erk2* ^−/−^ ES cells. (A) Western blot analysis of *Erk2*-null ES cells expressing exogenous 3X-FLAG-tagged ERK1 or ERK2. ES cells were cultured for 24 h in the presence of the FGF receptor inhibitor PD173074 (PD17) before wash out and stimulation with recombinant FGF2 (10 ng/ml) for 5–120 min. *3X-FLAG-ERK1 migrates slower than endogenous ERK1. **3X-FLAG-ERK2 co-migrates with ERK1. (B) qRT-PCR analysis of IEG induction following acute stimulation with recombinant FGF2 (10 ng/ml) in WT, *Erk2*-null, and *Erk2*-null ES cells rescued with either an *Erk1* or *Erk2* expression construct. As for the western blot analysis, cells were cultured for 24 h in PD17, followed by wash-out and stimulation with FGF2 for 1 h and 3 h. Robust FGF-mediated induction of *Egr2*, *Egr1*, and *cFos* was restored in *Erk2* ^−/−^ ES cells by either ERK1 or ERK2 overexpression. (C) Western blot analysis of lysates from wild-type (E14Ju), *Erk2*-null, and ERK2-rescued *Erk2*-null ES cells for β-catenin, pSTAT3, pAKT, and pSMAD2. No significant differences were observed in these signaling molecules. OCT4 and GAPDH were used as loading controls.

To test whether the attenuation of IEG induction in *Erk2*-null ES cells was isozyme specific, or a consequence of reduced total ERK activity, ERK2 depletion was rescued by transgenic expression of either 3xFLAG-ERK1 or 3xFLAG-ERK2. Western blot analysis showed that both exogenous ERK1 and ERK2 were expressed at comparable levels to endogenous ERK2 in WT cells, and were phosphorylated normally following FGF2 stimulation (Figure 4A). qRT-PCR analysis of IEG induction showed that either ERK isozyme was equally capable of reinstating normal levels of *Egr1*, *Egr2* and *cFos* induction (Figure 4B) arguing that ERK1 and ERK2 overlap functionally with respect to induction of this subset of IEGs in mouse ES cells. We also examined the activity of other signaling pathways and found that ERK2 deletion does not perturb the stability of β-catenin nor the phosphorylation status of STAT3, AKT, and SMAD2 in self-renewing ES cells (Figure 4C).

### ERK2 is Dispensable for Germ Layer Specification of ES Cells

**Figure 5:**
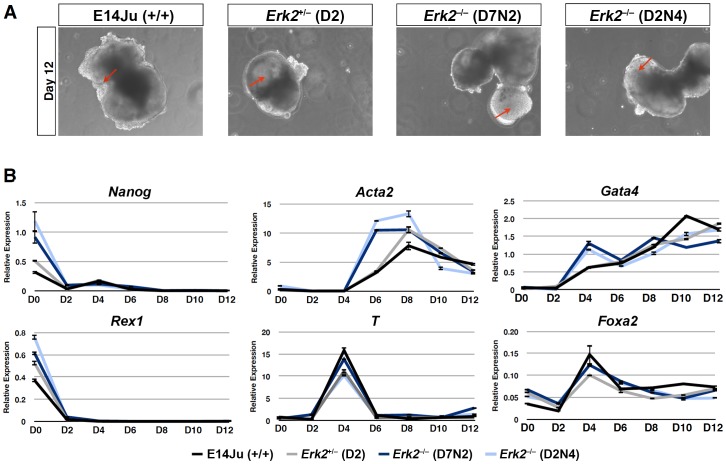
Germ layer differentiation of ES cells is ERK2 independent. (A) Phase contrast images of embryoid bodies (EBs) at 12 days of differentiation, arrow indicate presumptive cysts. (B) Time-course analysis of the progression of EB differentiation, showing similar differentiation kinetics in WT (E14Ju), heterozygous (D2) and homozygous (D7N2 and D2N4) Erk2 as shown by markers of pluripotency (*Oct4, Nanog*), mesoderm (*Acta2, T*), and endoderm (*Gata4, Foxa2*). Error bars represent the standard deviation from the mean of technical replicates.

We have previously reported a block in both neural and mesodermal differentiation in ERK2-deficient ES cells [18]. However, this block was not reversed upon transgenic rescue (Figure S2A,B), even though exogenous ERK2 was phosphorylated normally and rescued the defect in *Egr1* induction (Figure S2C,D). Moreover, ERK2-deficient cells were capable of generating both endodermal and mesodermal cell types in embryoid bodies (Figure S2E). Therefore it is likely that the differentiation defect exhibited by these ES cells is not related to the targeted mutation at the *Erk2* locus, and possibly related to genetic background, since they were derived from a C57Bl/6–129 F1 cross [23], or to an unknown mutation during the derivation of these ES cell lines from blastocysts. We therefore examined the ability of new *Erk2* ^−/−^ ES cells generated on a pure 129 background to commit to various differentiated lineages. Embryoid body (EB) differentiation was employed as it produces descendants of all three germ layers in 3-D culture, and is therefore an unbiased reflection of the potency of mES cells in culture. EBs produced from *Erk2* ^−/−^ ES cells exhibited similar morphological features to parental E14Ju wild-type ES cells (Figure 5A), with cystic EBs visible from day 6, and rhythmically beating cardiomyocytes evident by day 8 (data not shown). A time course of gene expression analysis showed no major differences in the down-regulation of the pluripotency markers, *Rex1* and *Nanog*, or up-regulation of endodermal (*Gata4*, *Foxa2*) and mesodermal markers (*T*, *Acta2*), indicating no general impairment in differentiation (Figure 5B). However, ERK2-deficient ES cells expressed higher levels of *Nanog* in self-renewing conditions (Day 0), and a more prominent induction of α-smooth muscle actin (*Acta2*) expression at Day 6 and *Gata4* at Day 4 (Figure 5B).

**Figure 6:**
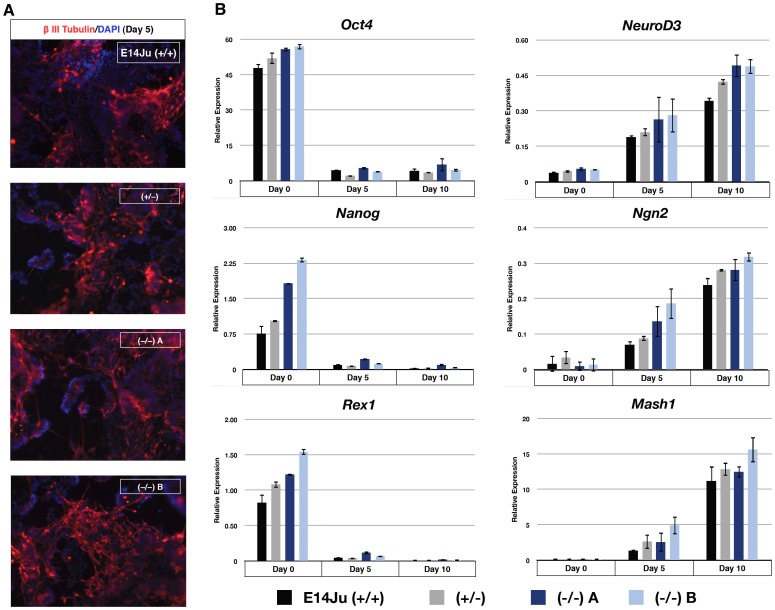
ERK2 is not required for ES cells to efficiently produce neurons under defined conditions. (A) Immunocytochemistry analysis of wild-type, *Erk2* ^+/−^ and *Erk2* ^−/−^ cultures after 5 days of neural induction for neuronal marker βIII Tubulin immunoreactivity (red). DNA is counterstained with DAPI in blue. (B) Gene expression analysis of cultures following 5 and 10 days of neural induction. Markers of pluripotency (*Oct4*, *Nanog*, and *Rex1*) and neural tissue (*NeuroD3, Ngn2*, and *Mash1*) are shown. Error bars represent the standard deviation from the mean of biological replicates.

Next we specifically investigated the requirement of ERK2 during neural induction using an established monolayer differentiation protocol in serum-free N2B27 medium [35], in which embryo-derived *Erk2* knock-out ES cells failed to form neural tissue (Figure S2) [18]. Following 5 days of differentiation ES-derived *Erk2*-null cells generated βIII tubulin-positive neurons with similar efficiency to WT and heterozygous lines (Figure 6A). qRT-PCR analysis showed almost complete down-regulation of pluripotency markers (*Oct4, Nanog, Rex1*) in all cell lines by day 5 of neural induction, with a concomitant increase in neural markers, *NeuroD3*, *Ngn2*, and *Mash1* (Figure 6B). As observed for the EB experiments, the expression of *Nanog* was higher in *Erk2*-null ES cells at Day 0 (Figure 6B), indicating that ES cells may self-renewal more efficiently in the absence of ERK2.

### ERK2 Suppresses Expression of a Subset of Pluripotent Genes

**Figure 7:**
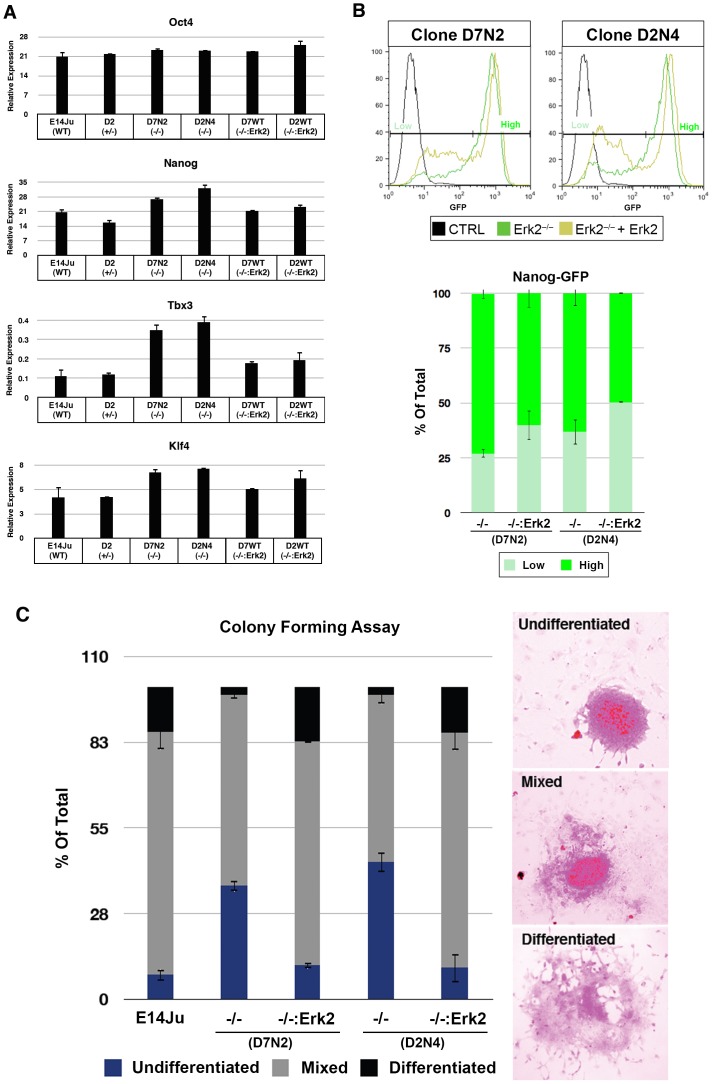
ERK2 deficiency results in increased expression of pluripotency markers and self-renewal capacity. (A) Gene expression analysis for pluripotency markers in two independent *Erk2* ^−/−^ ES cell clones, D7N2 and D2N4, and transgenic rescued subclones for each line, D7WT and D2WT. Error bars represent the standard deviation from the mean of 2 independent experiments. (B) Flow cytometry analysis of Nanog-GFP promoter activity of *Erk2*-null ES cells, and rescued subclones shows a shift towards lower Nanog-GFP expression in *Erk2*-rescued clones. (C) Clonal analysis in +LIF conditions showing enhanced self-renewal capacity of *Erk2*-null ES cells. Error bars represent the standard deviation from the mean of biological replicates.

To further investigate the elevated *Nanog* expression in ES cells lacking ERK2, two independent *Erk2* ^−/−^ ES cell clones were rescued with ERK2 expression constructs. Transgenic expression of ERK2 was capable of reinstating normal levels of *Nanog* to *Erk2* ^−/−^ ES cells (Figure 7A). A second elevated pluripotent marker, *Tbx3*, was similarly increased in *Erk2* ^−/−^ ES cells and rescued to wild-type levels by an ERK2 expression construct. A similar, but less dramatic effect was also observed for *Klf4* (Figure 7A). No significant difference was observed for *Oct4* levels across all cell lines tested. ERK2 deficiency had the most significant effect on *Tbx3* levels (a one-tailed t-test giving p-values of 0.02 and 0.01 for D7N2 and D2N4, respectively). This is consistent with previous reports that *Tbx3* is a negatively regulated direct target of ERK signaling in ES cells, and that TBX3 can positively regulate *Nanog* promoter activity [11]. It is of note that *Nanog*, *Tbx3*, *Klf4* are expressed heterogeneously in self-renewing ES cells [11] in contrast to the homogenous expression of *Oct4*, which did not exhibit significant differences in expression in the absence of ERK2. Therefore we hypothesized that ERK2 deficiency may reduce heterogeneity of self-renewing undifferentiated ES cells. To test this hypothesis, an EGFP cassette was knocked into the endogenous *Nanog* promoter in *Erk2* ^−/−^ ES cells (Figure S3) [36]. Two correctly targeted clones, one each from D7N2 and D2N4 *Erk2*-null lines, were stably transfected with an ERK2 expression cassette and the extent of EGFP expression was assessed by flow cytometry (Figure 7B). While the majority of *Erk2*-null ES cells exhibited high *Nanog*-GFP expression, a significant *Nanog*-GFP low subpopulation appeared when ERK2 was re-introduced (Figure 7B). This is consistent with other reports which show that Nanog heterogeneity is reduced in the presence of chemical inhibitors of MEK-ERK signaling [36], [37]. To further examine this phenomenon for a different pluripotent gene, we generated a *Tcfcp2l1*-Venus reporter construct and stably transfected it into *Erk2* ^−/−^ and ERK2-rescued *Erk2* ^−/−^ ES cells (Figure S4A). Tcfcp2l1 is a grainyhead transcription factor that is highly expressed in mouse ES cells and rapidly down-regulated upon differentiation [38], and it is a direct binding partner of OCT4 [39]. In self-renewing (+LIF) conditions 97% of *Erk2* ^−/−^ ES cells were positive for the *Tcfcp2l1*-Venus reporter, but this was reduced to 86% when ERK2 was added back to the cells (Figure S4B). The kinetics of *Tcfcp2l1*-Venus reporter expression during differentiation (removal of LIF) was examined for the *Erk2* ^−/−^ and ERK2-rescued ES cells and both cell lines efficiently down-regulated this reporter (Figure S4C). This result indicates that ERK2 plays a major role to generate heterogeneous gene expression in self-renewing ES cells, but has little impact on their down-regulation during differentiation.

### ERK2 Suppresses Self-renewal Capacity of ES Cells

To test if this reduced heterogeneity was functionally significant the self-renewal capacity of ERK2-deficient ES cells was tested in a stringent clonal density assay in the presence of LIF. Self-renewing cells were identified by alkaline phosphatase activity after 7 days at clonal density [40]. Colonies were counted and scored based on the degree of differentiation observed. In WT and *Erk2* ^+/−^ ES cells, a distribution of 10% undifferentiated, 70% mixed, and 20% differentiated colonies was observed (Figure 7C). This was in contrast to *Erk2*-null ES cell lines were a undifferentiated colonies increased from 10% to 30% and differentiated colonies were reduced from 20% to 5%. These percentages returned to a WT distribution upon transgenic expression of ERK2, indicating that ERK2 depletion enhances self-renewal in the presence of LIF. However, in the absence of LIF all genotypes differentiated to a similar extent (data not shown). Taken together these data indicate that loss of ERK2 increases the self-renewal capacity via reducing heterogeneous expression of key pluripotency genes in the presence of LIF, but is dispensable for lineage commitment of mouse ES cells. Our data supports a model where the total ERK activity, and not isozyme-specific activity, is critical for the balance between self-renewal and lineage commitment.

## Discussion

Here we have determined the consequences of *Erk2* deletion on FGF-ERK signal transduction, self-renewal capacity, and lineage specification in mouse ES cells. We have found that ES cells compensate for the loss of ERK2 by hyper-phosphorylation of the remaining ERK1 isozyme without an increase in *Erk1* transcription or translation. The mechanism may be through attenuation of induced negative feedback mechanisms, such as MAPK phosphatases, or direct feedback within the MAPK core module. We have found that immediate-early gene (IEG) induction is reduced in the absence of ERK2, as well as the negative regulator *Dusp6*. However, expression of a late response gene, *Ccnd2*, was unaffected by the loss of ERK2, underscoring the robustness of the ERK pathway to various perturbations [41]. Taken together this data suggests that ERK activity is stabilized, and maintained within a certain range, by both short (MAPK core cascade) and long (induced phosphatases) feedback loops and indicates that targeting these feedback loops may prove effective in modulating ERK activity in mES cells. To address the issue of redundancy we have shown that the early transcriptional defects in response to FGF activation observed in *Erk2*-null ES cells is entirely rescued by expression of either ERK1 or ERK2 implying that in ES cells it is total ERK activity, rather than isozyme specificity, that is most important for IEG induction.

In contrast to blastocyst-derived *Erk2* ^−/−^ ES cells of mixed genetic background, *Erk2* deletion by two rounds of homologous recombination in ES cells of a uniform genetic background (129/Ola) has no detectable effect on lineage specification into either neural, endodermal, or mesodermal lineages, and in fact ERK2-deficient ES cells appeared to differentiate towards the mesoderm lineage more efficiently that either WT or heterozygous lines, as judged by increased induction of *Gata4* and *Acta2* expression (Figure 5B).

The lack of lineage commitment defects we observe is in agreement with recent embryological data indicating that ERK2 is not required for lineage specification in the embryo proper. Voisin and colleagues used tetraploid complementation, where the extra-embryonic tissues are wild-type, to produce grossly normal E13.5 *Erk2* ^−/−^ embryos [42]. As reported previously, the early phenotype of *Erk2*-null embryos was due to cell autonomous defects in the trophoblast lineage [23], and that the reported lack in mesoderm induction [24], is likely a secondary defect due to a lack of inductive signals from a defective extraembryonic ectoderm.

Elevated expression of key pluripotency-associated genes, *Nanog*, *Tbx3*, and *Klf4* was detected in *Erk2*-null ES cells when compared to WT or ERK2-rescued cells. The *Nanog*-GFP reporter and *Tcfcp2l1*-Venus reporter on the *Erk2*-null background suggests this increase in expression is probably due to reduced heterogeneity in ES cell cultures. Undifferentiated *Nanog*-GFP-low ES cells are more prone to differentiate than *Nanog*-GFP-high ES cells [36], and increased self-renewal activity of *Erk2*-null ES cells in stringent clonal assays is consistent with their reduced heterogeneity. Recent work has shown that ERK activity in ES cells increases the heterogeneous expression of the primitive endoderm marker, *Hex*, and epiblast marker, *Tcf15* [43], [44]. These results suggest that lowering the total active ERK pool in ES cells reduces fluctuation of lineage-priming genes and parts of the pluripotency transcriptional network, consistent with observations that chemical inhibition of the MEK-ERK pathway promotes self-renewal and inhibits lineage commitment [5], [45]. The increase in *Nanog* and *Tbx3* expression observed in *Erk2* ^−/−^ ES cells may contribute to the enhanced performance in clonal assays, since over-expression of either gene strongly promotes the self-renewal capacity of ES cells [11], [40]. In summary, this work reveals that mouse ES cells can cope with the removal of ERK2 partially by up-regulating the activity of ERK1. This apparent functional redundancy between the ERK enzymes has been clearly shown in embryonic fibroblasts, where *Erk1* ^−/−^;*Erk2* ^−/−^ cells rapidly undergo senescence [42]. The complete genetic removal of both *Erk1* and *Erk2* from ES cells will be necessary to determine if naïve mouse pluripotent stem cells require ERK enzymes for cell survival, self-renewal, and to efficiently exit self-renewal to generate committed lineages.

## Materials and Methods

### Targeting Vectors and Expression Constructs

*Erk2* targeting vectors were constructed with *Erk2* homology arms kindly provided by S. Meloche and M. Saba-El-Leil (30). Homology arms were digested with BglII to remove a 2.5 kb fragment flanking exon 3. The vector backbone, containing 4.8 kb 5′ homology and 2.8 kb 3′ homology, was then ligated to a BamHI fragment containing the floxed CMV-Hph-TK cassette. To this a diphtheria toxin A cassette was added outside of the homology arms as a HincII fragment. The second targeting construct was generated by replacing the CMV-Hph-TK cassette with a BamHI fragment containing a mPGK-Neo^r^ cassette. ERK1 and ERK2 expression constructs were generated by amplifying *Erk1* and *Erk2* coding regions from E14Tg2a ES cell cDNA with primers containing NotI and BamHI sites. The resulting PCR fragments were digest and ligated in-frame with the 3xFLAG epitope contained in a pCAG-driven expression vector with puromycin-resistance [46]. The *Tcfcp2l1*-Venus reporter construct was constructed as follows. An 8.5 kb SpeI fragment was taken from a BAC clone (bMQ332b16) which contained a putative promoter region, exon 1, and most of intron 1 of the *Tcfcp2l1* gene. This was cloned upstream of an adenovirus splice acceptor (SA) and an internal ribosome entry site (IRES) from the gene-trap vector pGT1V3 which contained a Venus expression cassette [47]. A PEST sequence was added to the C-terminal of Venus to decrease its stability [48] and increase the sensitivity of the promoter. A second IRES from the encephalomyocarditis virus (ECMV) [49] was added to link expression of the puromycin N-acetyl-transferase (PAC) gene for selection of Venus expressing clones (Figure S4A). Vector sequence is available upon request.

### ES Cell Culture and Gene Targeting

All ES cell lines were maintained in complete ES cell medium: GMEM (Sigma, G5154) supplemented with 10% FBS (Invitrogen), 100 µM 2-mercaptoethanol (BDH, 441413), 1xMEM non-essential amino acids (Invitrogen, 1140-036), 2 mM L-glutamine, 1 mM sodium pyruvate (both from Invitrogen), and 1000 units/ml LIF (made in-house) on gelatinised tissue culture flasks (Iwaki). The FGFR and Mek1 inhibitors, PD173074 and PD0325901, were used at 200 nM and 1 µM, respectively, and were both from Axon Medchem. All *Erk2* gene targeting was performed in E14Ju ES cells, which is a subclone of E14Tg2a ES cells [50]. E14Ju ES cells (10^6^) were electroporated with linearized DNA (100 µg) using a BioRad GenePulser (0.8 kV and 10 µF). Stable transfectants were selected for with the appropriate antibiotic: Hygromycin (100 µg/ml); G418 (150 µg/ml); Puromycin (1 µg/ml). ES cell colonies were picked to 96-well plates, replica plated, and initial screening for targeting was by qPCR of genomic DNA for exon 3 of *Erk2*. Exon 3 primers are forward: 5′-CTAACGTTCTGCACCGTGAC-3′ and reverse: 5′-GATCACAAGTGGTGTTCAGCAG-3′, used with Roche Universal Probe Library (UPL) probe #97 and control primers for diploidy were for *Spry1* forward: 5′-CTAACGTTCTGCACCGTGAC-3′ and reverse: 5′-GATCACAAGTGGTGTTCAGCAG-3′ used with UPL probe #78. The EGFP-IRES-Puro^R^ cassette was targeted to the *Nanog* locus in *Erk2* ^−/−^ ES cells as previously described [36].

### Southern Blotting

Southern blotting was performed by resolving 5 µg gDNA, digested with KpnI alone or KpnI-PstI, by agarose gel electrophoresis. gDNA was stained with ethidium bromide and nicked using a UV trans-illuminator, followed by denaturation in 500 mM NaOH, 1.5 M NaCl for 1 hour. The reaction was stopped by incubating the gel in 500 mM Tris, 1.5 mM NaCl, (pH 5.5) and gDNA was transferred to a nitrocellulose membrane (Amersham) overnight by capillary action in the presence of 20X SSC. The DNA was then cross-linked to the membrane using a UV oven (Stratalink) and baked for 1 h at 120°C. The hybridization probe was labelled using the MegaPrime™ DNA Labeling System (Amersham) according to manufacturer’s instructions, using [α^−32^P] dCTP, 3000 Ci/mmol and incubated overnight at 68°C. Following extensive washes the membrane was exposed to X-ray film (Kodak) overnight at −80°C and subsequently developed in an automated autoradiographic film processor.

### ES Cell Stimulation, Differentiation and Clonal Assays

For acute FGF2 simulation, ES cells were plated at 2×10^4^ cells/cm^2^ in complete ES cell medium. The following day inhibitors were added (PD173074, 100 nM or PD0325901, 1 µM, both from Axon Medchem) and cultured for a further 24 hours. Inhibitors were washed out with PBS (2×2 ml) and cells were stimulated with complete ES cell medium supplemented with recombinant hFGF2 (10 ng/ml, NCI Frederick BRB Repository) for 5 min to 3 hours. Serum-free neural induction was performed as described by Ying et al. with N2B27 medium made in-house [35]. Embryoid body differentiation was performed in hanging drops of ES cell medium in the absence of LIF. Cells were diluted to a concentration of 3.2×10^3^ cells/ml and aliquoted in 30 µl drops. After 2 days the aggregates were transferred to low adhesion bacterial grade culture dishes and cultured for the indicated times. Self-renewal capability was determined in a colony-forming assay by plating at low density (0.2×10^3^ cells/cm^2^) in the presence or absence of LIF. Colonies were allowed to form over 7 days whereupon they were fixed, stained for alkaline phosphatase activity using the Leukocyte Alkaline Phosphatase Assay Kit (Sigma), and classified as either (i) undifferentiated, (ii) mixed, or (iii) differentiated.

### Western Blotting

Western blotting and multi-strip blotting were performed as described previously [18], [51]. Membranes were probed with rabbit anti-total ERK1/2 #4695 (1∶1000), rabbit anti-phospho-ERK1/2 #4370 (1∶2000), rabbit anti-phospho-RSK T359/S363 #9344 (1∶1000), rabbit anti-total RSK #9355 (1∶1000), rabbit anti-phospho-SMAD2 #3108 (1∶1000), rabbit anti-phospho-MEK1/2 #9154, mouse anti-MEK1/2 #4694 (1∶1000), rabbit anti-phospho-AKT #4060, and rabbit anti-phospho-STAT3 #9145, all from Cell Signaling, anti-Tubulin (1∶2000, Abcam), mouse anti-GAPDH (1∶35000, Sigma), mouse anti-OCT4 (1∶500, SCBT), and rabbit anti-β-Catenin (1∶1000, Sigma) and detected with the appropriate HRP conjugated secondary (Pierce) and peroxidase activity was visualised with the SuperSignal West Pico Kit (Pierce). Membranes were stripped with 100 mM 2-mercaptoethanol in 72.5 mM Tris (pH 7.6) at 60°C for 15 minutes, and sequentially probed with the indicated antibodies. For quantitative western blotting, primary antibodies were detected with secondary antibodies conjugated to Alexa fluorophores (Molecular Probes, 1∶5000) and visualized using the BioRad ChemiDoc MP system. Images were quantified based on pixel density using the ImageJ software package.

### Quantitative Real-time Polymerase Chain Reaction (qRT-PCR)

Total RNA was extracted using Trizol reagent (Invitrogen) according to the manufacturer’s instructions. First-strand synthesis was performed using M-MLV RT (Invitrogen) with 50 ng random hexamers (Thermo Fisher) in the presence of RNase inhibitor, RNaseOUT (Invitrogen), in a reaction volume of 20 µl. Following first-strand synthesis the reaction mixture was diluted to 80 µl, and 2 µl was used per qPCR reaction in Probes Master Mix (Roche) using Universal Probe Library (UPL) probes and the LightCycler 480 system (Roche). Concentrations were determined against a standard curve and values normalized to *TBP* expression, as a house-keeping gene. Primers sequences and UPL probe numbers are available in Supporting Table S1.

### Immunocytochemistry

Cells were fixed in 4% paraformaldehyde (w/v) at room temperature for 15 minutes followed by single step permeabilization and blocking in 0.1% Triton-X and 2% goat serum for 1 hour. Cells were incubated with anti-βIII Tubulin (TuJ1, 1∶1000, R&D Systems) overnight. Following extensive washes, secondary antibodies conjugated to Alexa fluorophores (Molecular Probes) were diluted 1∶1000 in blocking buffer and applied for 1 hour at room temperature. The cells were washed twice in PBS and a third time in PBS containing DAPI (10 µg/ml) before obtaining pictures on an Olympus IX51 inverted fluorescence microscope.

### Flow Cytometry

Cells were collected by trypsinization and resuspended in PBS supplemented with 3% FBS. Samples were analyzed using a FACSCalibur flow cytometer (BD Biosystems) and data processed with FlowJo (Tree Star Inc.).

## Supporting Information

Figure S1 **Normal ploidy of** ***Erk2*** **-null ES cell lines.** Metaphase spreads of four *Erk2*-null ES cell lines were stained with Giemsa and chromosome counts indicated largely normal ploidy.
(TIF)

Figure S2 **Embryo-derived** ***Erk2*** **^−/−^ ES cell lines exhibit an ERK2-independent block in lineage specification.** (A) Immunocytochemistry analysis of wild-type (E14Tg2a), embryo-derived *Erk2* ^−/−^ (B1) ES cells, and ERK2-rescued *Erk2* ^−/−^ ES cells following 7 days of neural induction for neuronal marker βIII Tubulin. DNA is counterstained with DAPI. (B) qRT-PCR analysis of markers of pluripotency (*Oct4*), and neural tissue (*Nestin*, βIII Tubulin, also known as *Tubb3*). (C) Western blot analysis of E14Tg2a and *Erk2* ^−/−^ +3X-FLAG-ERK2 showing phosphorylation of exogenously expressed ERK2. (D) qRT-PCR analysis of FGF2-mediated induction of *Egr1* in E14Tg2a, embryo-derived *Erk2* ^−/−^ ES cells, and ERK2-rescued *Erk2* ^−/−^ ES cells. Transgenic add-back of ERK2 efficiently rescues normal *Egr1* expression to *Erk2* ^−/−^ ES cells in three independent clones. (E) qRT-PCR analysis of embryoid bodies at 6 days of differentiation for markers of pluripotency (*Nanog* and *Oct4*), mesoderm (*Pdgfrα*), and endoderm (*Gata6*).
(TIF)

Figure S3 ***Nanog*** **-GFP targeting.** (A) Schematic depicting *Nanog* targeting strategy. Black bars denote the position of the 5′ external probe used for Southern analysis. (B) Southern blot analysis of SexAI-digested genomic DNA showing correct targeting in 9 of 10 clones tested. D2TNG and D7TNG clones were derived from D2N4 and D7N2 *Erk2*-null lines, respectively.
(TIF)

Figure S4 ***Tcfcp2l1*** **-Venus reporter shows increased expression in** ***Erk2*** **^−/−^ ES cells.** (A) Schematic (not to scale) depicting the final *Tcfcp2l1*-Venus reporter construct. An 8.5 kb fragment of the mouse *Tcfcp2l1* gene was cloned upstream of a splice-acceptor (SA) and GTX-IRES (IRES1), followed by a destabilized Venus protein (Venus-PEST), an ECMV-IRES (IRES2), a puromycin-resistance gene (PAC), and a polyadenylation signal (pA). (B) FACS analysis of *Erk2* ^−/−^ ES cells and ERK2-rescued *Erk2* ^−/−^ ES cells with the *Tcfcp2l1*-Venus reporter showed that Venus expression in self-renewing conditions was highest in the absence of ERK2. (C) Down-regulation of Venus expression over 48 h of differentiation followed similar kinetics in the presence or absence of ERK2.
(TIF)

Table S1 **PCR primers for qRT-PCR analysis.** Forward (F) and reverse (R) primer sequences (5′-3′) and Roche Universal Probe Library (UPL) probes used in qRT-PCR assays.
(PDF)

## References

[1] Brook FA, Gardner RL (1997) The origin and efficient derivation of embryonic stem cells in the mouse. Proc Natl Acad Sci USA 94: 5709–5712. 9159137 10.1073/pnas.94.11.5709 PMC20843

[2] Evans MJ, Kaufman MH (1981) Establishment in culture of pluripotential cells from mouse embryos. Nature 292: 154–156. 7242681 10.1038/292154a0

[3] Martin GR (1981) Isolation of a pluripotent cell line from early mouse embryos cultured in medium conditioned by teratocarcinoma stem cells. Proc Natl Acad Sci USA 78: 7634–7638. 6950406 10.1073/pnas.78.12.7634 PMC349323

[4] Robertson E, Bradley A, Kuehn M, Evans M (1986) Germ-line transmission of genes introduced into cultured pluripotential cells by retroviral vector. Nature 323: 445–448 doi:10.1038/323445a0. 3762693 10.1038/323445a0

[5] Ying Q-L, Wray J, Nichols J, Batlle-Morera L, Doble B, et al (2008) The ground state of embryonic stem cell self-renewal. Nature 453: 519–523 doi:10.1038/nature06968. 18497825 10.1038/nature06968 PMC5328678

[6] Najm FJ, Chenoweth JG, Anderson PD, Nadeau JH, Redline RW, et al (2011) Isolation of epiblast stem cells from preimplantation mouse embryos. Cell Stem Cell 8: 318–325 doi:10.1016/j.stem.2011.01.016. 21362571 10.1016/j.stem.2011.01.016 PMC3073125

[7] Ying Q-L, Nichols J, Chambers I, Smith A (2003) BMP induction of Id proteins suppresses differentiation and sustains embryonic stem cell self-renewal in collaboration with STAT3. Cell 115: 281–292. 14636556 10.1016/s0092-8674(03)00847-x

[8] Avilion AAA, Nicolis SKS, Pevny LHL, Perez LL, Vivian NN, et al (2003) Multipotent cell lineages in early mouse development depend on SOX2 function. Genes Dev 17: 126–140 doi:10.1101/gad.224503. 12514105 10.1101/gad.224503 PMC195970

[9] Niwa H, Miyazaki J, Smith AG (2000) Quantitative expression of Oct-3/4 defines differentiation, dedifferentiation or self-renewal of ES cells. Nat Genet 24: 372–376 doi:10.1038/74199. 10742100 10.1038/74199

[10] Loh Y-H, Wu Q, Chew J-L, Vega VB, Zhang W, et al (2006) The Oct4 and Nanog transcription network regulates pluripotency in mouse embryonic stem cells. Nat Genet 38: 431–440 doi:10.1038/ng1760. 16518401 10.1038/ng1760

[11] Niwa H, Ogawa K, Shimosato D, Adachi K (2009) A parallel circuit of LIF signalling pathways maintains pluripotency of mouse ES cells. Nature 460: 118–122 doi:10.1038/nature08113. 19571885 10.1038/nature08113

[12] Silva J, Smith A (2008) Capturing pluripotency. Cell 132: 532–536 doi:10.1016/j.cell.2008.02.006. 18295569 10.1016/j.cell.2008.02.006 PMC2427053

[13] Arman E, Haffner-Krausz R, Chen Y, Heath JK, Lonai P (1998) Targeted disruption of fibroblast growth factor (FGF) receptor 2 suggests a role for FGF signaling in pregastrulation mammalian development. Proc Natl Acad Sci USA 95: 5082–5087. 9560232 10.1073/pnas.95.9.5082 PMC20217

[14] Chazaud C, Yamanaka Y, Pawson T, Rossant J (2006) Early lineage segregation between epiblast and primitive endoderm in mouse blastocysts through the Grb2-MAPK pathway. Developmental Cell 10: 615–624 doi:10.1016/j.devcel.2006.02.020. 16678776 10.1016/j.devcel.2006.02.020

[15] Cheng AM, Saxton TM, Sakai R, Kulkarni S, Mbamalu G, et al (1998) Mammalian Grb2 regulates multiple steps in embryonic development and malignant transformation. Cell 95: 793–803. 9865697 10.1016/s0092-8674(00)81702-x

[16] Feldman B, Poueymirou W, Papaioannou VE, DeChiara TM, Goldfarb M (1995) Requirement of FGF-4 for postimplantation mouse development. Science 267: 246–249. 7809630 10.1126/science.7809630

[17] Yuan H, Corbi N, Basilico C, Dailey L (1995) Developmental-specific activity of the FGF-4 enhancer requires the synergistic action of Sox2 and Oct-3. Genes Dev 9: 2635–2645. 7590241 10.1101/gad.9.21.2635

[18] Kunath T, Saba-El-Leil MK, Almousailleakh M, Wray J, Meloche S, et al (2007) FGF stimulation of the Erk1/2 signalling cascade triggers transition of pluripotent embryonic stem cells from self-renewal to lineage commitment. Development 134: 2895–2902 doi:10.1242/dev.02880. 17660198 10.1242/dev.02880

[19] Wilder PJ, Kelly D, Brigman K, Peterson CL, Nowling T, et al (1997) Inactivation of the FGF-4 gene in embryonic stem cells alters the growth and/or the survival of their early differentiated progeny. Dev Biol 192: 614–629 doi:10.1006/dbio.1997.8777. 9441693 10.1006/dbio.1997.8777

[20] Nichols J, Jones K, Phillips JM, Newland SA, Roode M, et al (2009) Validated germline-competent embryonic stem cell lines from nonobese diabetic mice. Nat Med 15: 814–818 doi:10.1038/nm.1996. 19491843 10.1038/nm.1996

[21] Buehr M, Meek S, Blair K, Yang J, Ure J, et al (2008) Capture of authentic embryonic stem cells from rat blastocysts. Cell 135: 1287–1298 doi:10.1016/j.cell.2008.12.007. 19109897 10.1016/j.cell.2008.12.007

[22] Li P, Tong C, Mehrian-Shai R, Jia L, Wu N, et al (2008) Germline competent embryonic stem cells derived from rat blastocysts. Cell 135: 1299–1310 doi:10.1016/j.cell.2008.12.006. 19109898 10.1016/j.cell.2008.12.006 PMC2735113

[23] Saba-El-Leil MK, Vella FDJ, Vernay B, Voisin L, Chen L, et al (2003) An essential function of the mitogen-activated protein kinase Erk2 in mouse trophoblast development. EMBO Rep 4: 964–968 doi:10.1038/sj.embor.embor939. 14502223 10.1038/sj.embor.embor939 PMC1326397

[24] Yao Y, Li W, Wu J, Germann UA, Su MSS, et al (2003) Extracellular signal-regulated kinase 2 is necessary for mesoderm differentiation. Proc Natl Acad Sci USA 100: 12759–12764 doi:10.1073/pnas.2134254100. 14566055 10.1073/pnas.2134254100 PMC240691

[25] Fischer AM, Katayama CD, Pagès G, Pouysségur J, Hedrick SM (2005) The role of erk1 and erk2 in multiple stages of T cell development. Immunity 23: 431–443 doi:10.1016/j.immuni.2005.08.013. 16226508 10.1016/j.immuni.2005.08.013

[26] Dalby KN, Morrice N, Caudwell FB, Avruch J, Cohen P (1998) Identification of regulatory phosphorylation sites in mitogen-activated protein kinase (MAPK)-activated protein kinase-1a/p90rsk that are inducible by MAPK. J Biol Chem 273: 1496–1505. 9430688 10.1074/jbc.273.3.1496

[27] Santos SDM, Verveer PJ, Bastiaens PIH (2007) Growth factor-induced MAPK network topology shapes Erk response determining PC-12 cell fate. Nat Cell Biol 9: 324–330 doi:10.1038/ncb1543. 17310240 10.1038/ncb1543

[28] De Cesare D, Jacquot S, Hanauer A, Sassone-Corsi P (1998) Rsk-2 activity is necessary for epidermal growth factor-induced phosphorylation of CREB protein and transcription of c-fos gene. Proc Natl Acad Sci USA 95: 12202–12207. 9770464 10.1073/pnas.95.21.12202 PMC22809

[29] Nakakuki T, Birtwistle MR, Saeki Y, Yumoto N, Ide K, et al (2010) Ligand-specific c-Fos expression emerges from the spatiotemporal control of ErbB network dynamics. Cell 141: 884–896 doi:10.1016/j.cell.2010.03.054. 20493519 10.1016/j.cell.2010.03.054 PMC2888034

[30] Choudhury GG (2001) Akt serine threonine kinase regulates platelet-derived growth factor-induced DNA synthesis in glomerular mesangial cells: regulation of c-fos AND p27(kip1) gene expression. J Biol Chem 276: 35636–35643 doi:10.1074/jbc.M100946200. 11470779 10.1074/jbc.M100946200

[31] Brondello JM, Pouysségur J, McKenzie FR (1999) Reduced MAP kinase phosphatase-1 degradation after p42/p44MAPK-dependent phosphorylation. Science 286: 2514–2517. 10617468 10.1126/science.286.5449.2514

[32] Brondello JM, Brunet A, Pouysségur J, McKenzie FR (1997) The dual specificity mitogen-activated protein kinase phosphatase-1 and -2 are induced by the p42/p44MAPK cascade. J Biol Chem 272: 1368–1376. 8995446 10.1074/jbc.272.2.1368

[33] Murphy LO, Smith S, Chen R-H, Fingar DC, Blenis J (2002) Molecular interpretation of ERK signal duration by immediate early gene products. Nat Cell Biol 4: 556–564 doi:10.1038/ncb822. 12134156 10.1038/ncb822

[34] White J, Dalton S (2005) Cell cycle control of embryonic stem cells. Stem Cell Rev 1: 131–138 doi:10.1385/SCR:1:2:131. 17142847 10.1385/SCR:1:2:131

[35] Ying Q-L, Stavridis M, Griffiths D, Li M, Smith A (2003) Conversion of embryonic stem cells into neuroectodermal precursors in adherent monoculture. 4 doi:10.1038/nbt780. 10.1038/nbt780 12524553

[36] Chambers I, Silva J, Colby D, Nichols J, Nijmeijer B, et al (2007) Nanog safeguards pluripotency and mediates germline development. Nature 450: 1230–1234 doi:10.1038/nature06403. 18097409 10.1038/nature06403

[37] Singh AM, Hamazaki T, Hankowski KE, Terada N (2007) A heterogeneous expression pattern for Nanog in embryonic stem cells. Stem Cells 25: 2534–2542 doi:10.1634/stemcells.2007-0126. 17615266 10.1634/stemcells.2007-0126

[38] Ivanova N, Dobrin R, Lu R, Kotenko I, Levorse J, et al (2006) Dissecting self-renewal in stem cells with RNA interference. Nature 442: 533–538 doi:10.1038/nature04915. 16767105 10.1038/nature04915

[39] van den Berg DLC, Snoek T, Mullin NP, Yates A, Bezstarosti K, et al (2010) An Oct4-centered protein interaction network in embryonic stem cells. Cell Stem Cell 6: 369–381 doi:10.1016/j.stem.2010.02.014. 20362541 10.1016/j.stem.2010.02.014 PMC2860243

[40] Chambers I, Colby D, Robertson M, Nichols J, Lee S, et al (2003) Functional expression cloning of Nanog, a pluripotency sustaining factor in embryonic stem cells. Cell 113: 643–655. 12787505 10.1016/s0092-8674(03)00392-1

[41] Kholodenko BN, Hancock JF, Kolch W (2010) Signalling ballet in space and time. Nat Rev Mol Cell Biol 11: 414–426 doi:10.1038/nrm2901. 20495582 10.1038/nrm2901 PMC2977972

[42] Voisin L, Saba-El-Leil MK, Julien C, Frémin C, Meloche S (2010) Genetic demonstration of a redundant role of extracellular signal-regulated kinase 1 (ERK1) and ERK2 mitogen-activated protein kinases in promoting fibroblast proliferation. Mol Cell Biol 30: 2918–2932 doi:10.1128/MCB.00131-10. 20368360 10.1128/MCB.00131-10 PMC2876689

[43] Canham MA, Sharov AA, Ko MSH, Brickman JM (2010) Functional heterogeneity of embryonic stem cells revealed through translational amplification of an early endodermal transcript. PLoS Biol 8: e1000379 doi:10.1371/journal.pbio.1000379. 20520791 10.1371/journal.pbio.1000379 PMC2876051

[44] Davies OR, Lin C-Y, Radzisheuskaya A, Zhou X, Taube J, et al (2013) Tcf15 primes pluripotent cells for differentiation. Cell Rep 3: 472–484 doi:10.1016/j.celrep.2013.01.017. 23395635 10.1016/j.celrep.2013.01.017 PMC3607254

[45] Burdon T, Stracey C, Chambers I, Nichols J, Smith A (1999) Suppression of SHP-2 and ERK signalling promotes self-renewal of mouse embryonic stem cells. Dev Biol 210: 30–43 doi:10.1006/dbio.1999.9265. 10364425 10.1006/dbio.1999.9265

[46] Niwa H, Yamamura K, Miyazaki J (1991) Efficient selection for high-expression transfectants with a novel eukaryotic vector. Gene 108: 193–199. 1660837 10.1016/0378-1119(91)90434-d

[47] Tsakiridis A, Tzouanacou E, Rahman A, Colby D, Axton R, et al (2009) Expression-independent gene trap vectors for random and targeted mutagenesis in embryonic stem cells. Nucleic Acids Res 37: e129 doi:10.1093/nar/gkp640. 19692586 10.1093/nar/gkp640 PMC2770648

[48] Rogers S, Wells R, Rechsteiner M (1986) Amino acid sequences common to rapidly degraded proteins: the PEST hypothesis. Science 234: 364–368. 2876518 10.1126/science.2876518

[49] Gurtu V, Yan G, Zhang G (1996) IRES bicistronic expression vectors for efficient creation of stable mammalian cell lines. Biochemical and Biophysical Research Communications 229: 295–298 doi:10.1006/bbrc.1996.1795. 8954121 10.1006/bbrc.1996.1795

[50] Hooper M, Hardy K, Handyside A, Hunter S, Monk M (1987) HPRT-deficient (Lesch-Nyhan) mouse embryos derived from germline colonization by cultured cells. Nature 326: 292–295 doi:10.1038/326292a0. 3821905 10.1038/326292a0

[51] Kiyatkin A, Aksamitiene E (2009) Multistrip western blotting to increase quantitative data output. Methods Mol Biol 536: 149–161 doi:10.1007/978-1-59745-542-8-17. 19378054 10.1007/978-1-59745-542-8_17 PMC2923389

