# Supplementary figures and images for: ERK2 Suppresses Self-Renewal Capacity of Embryonic Stem Cells, but Is Not Required for Multi-Lineage Commitment
Source: PLoS One. 2013 Apr 16;8(4):e60907. doi: 10.1371/journal.pone.0060907 (PMC3628700; doi:10.1371/journal.pone.0060907)

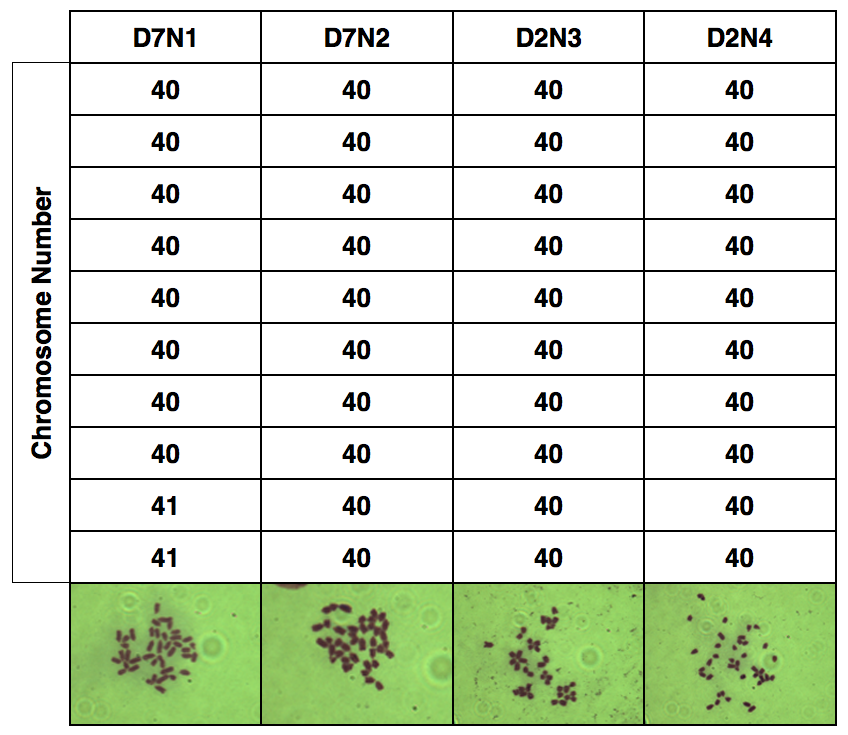

Supplement: Figure S1 — Normal ploidy of Erk2 -null ES cell lines. Metaphase spreads of four Erk2-null ES cell lines were stained with Giemsa and chromosome counts indicated largely normal ploidy. (TIF) [file pone.0060907.s001.tif]

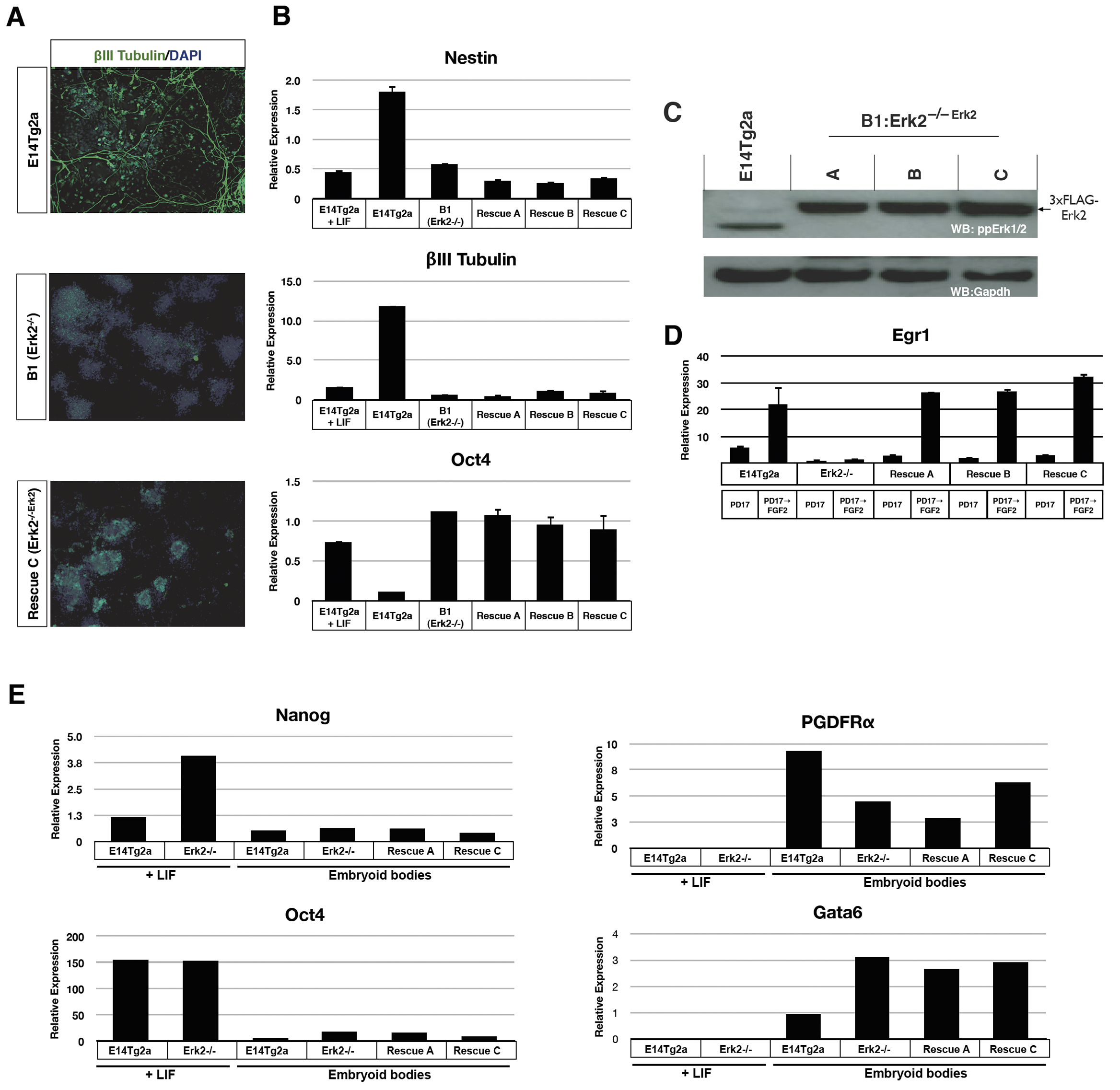

Supplement: Figure S2 — Embryo-derived Erk2 −/− ES cell lines exhibit an ERK2-independent block in lineage specification. (A) Immunocytochemistry analysis of wild-type (E14Tg2a), embryo-derived Erk2 −/− (B1) ES cells, and ERK2-rescued Erk2 −/− ES cells following 7 days of neural induction for neuronal marker βIII Tubulin. DNA is counterstained with DAPI. (B) qRT-PCR analysis of markers of pluripotency (Oct4), and neural tissue (Nestin, βIII Tubulin, also known as Tubb3). (C) Western blot analysis of E14Tg2a and Erk2 −/− +3X-FLAG-ERK2 showing phosphorylation of exogenously expressed ERK2. (D) qRT-PCR analysis of FGF2-mediated induction of Egr1 in E14Tg2a, embryo-derived Erk2 −/− ES cells, and ERK2-rescued Erk2 −/− ES cells. Transgenic add-back of ERK2 efficiently rescues normal Egr1 expression to Erk2 −/− ES cells in three independent clones. (E) qRT-PCR analysis of embryoid bodies at 6 days of differentiation for markers of pluripotency (Nanog and Oct4), mesoderm (Pdgfrα), and endoderm (Gata6). (TIF) [file pone.0060907.s002.tif]

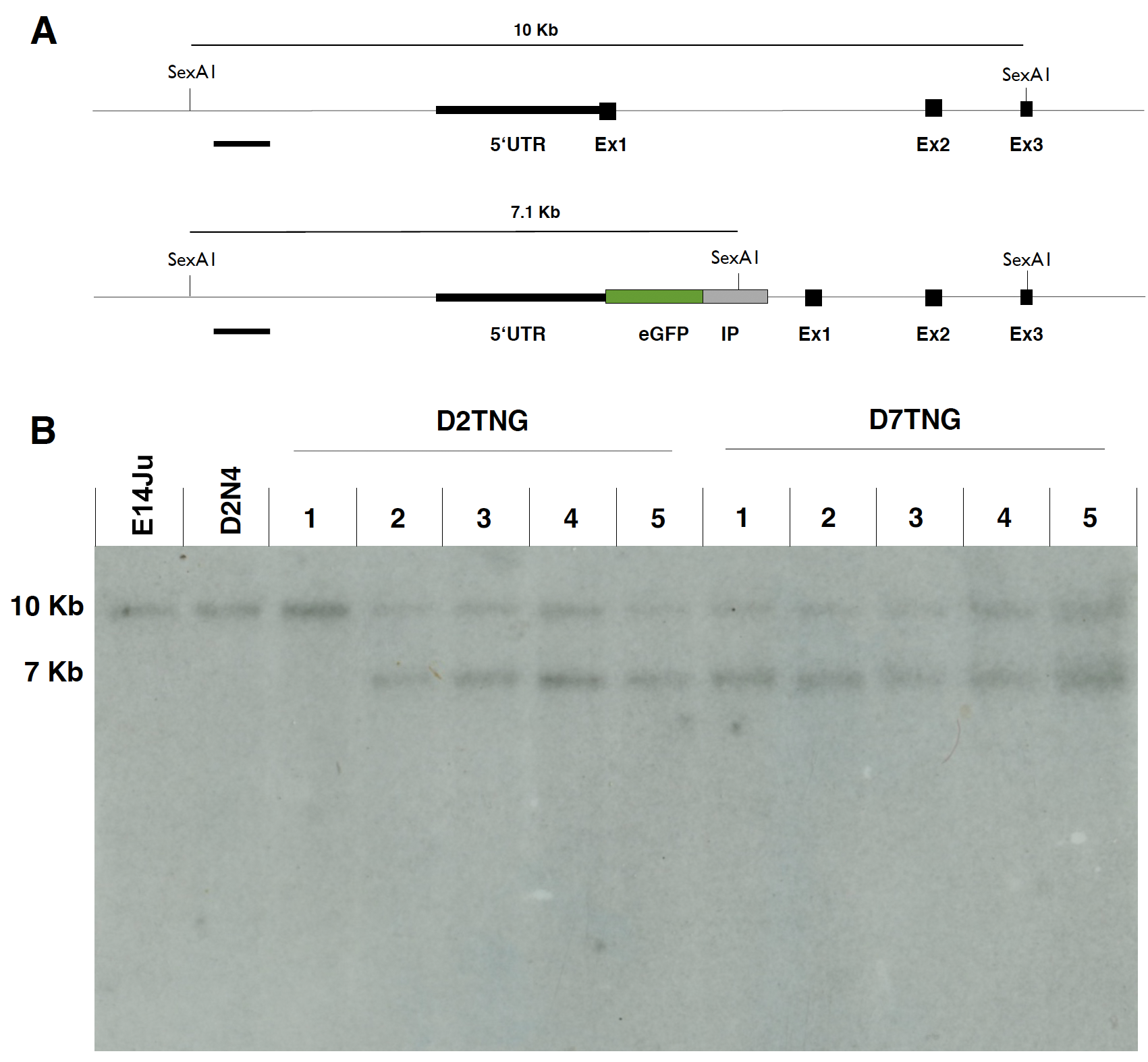

Supplement: Figure S3 — Nanog -GFP targeting. (A) Schematic depicting Nanog targeting strategy. Black bars denote the position of the 5′ external probe used for Southern analysis. (B) Southern blot analysis of SexAI-digested genomic DNA showing correct targeting in 9 of 10 clones tested. D2TNG and D7TNG clones were derived from D2N4 and D7N2 Erk2-null lines, respectively. (TIF) [file pone.0060907.s003.tif]

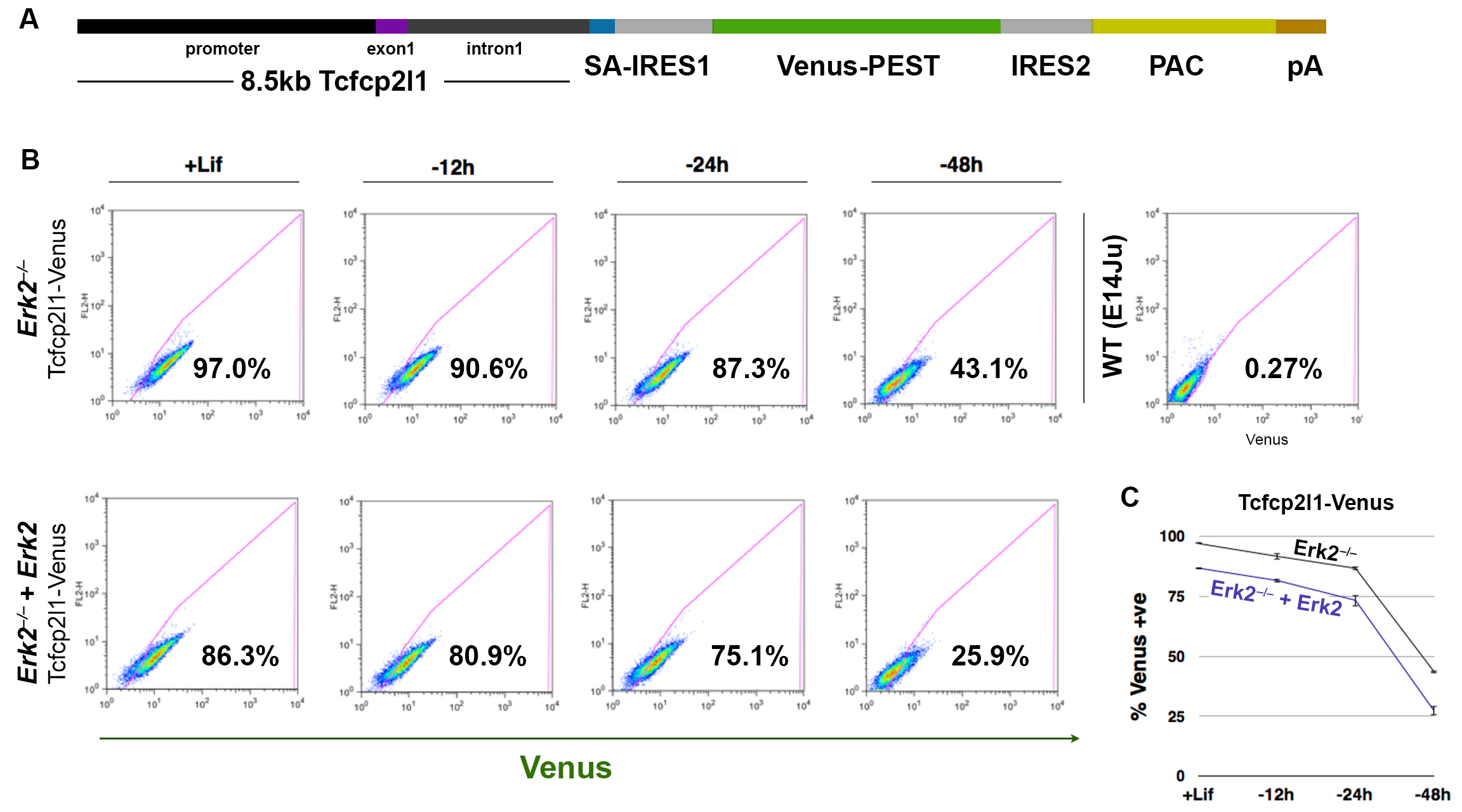

Supplement: Figure S4 — Tcfcp2l1 -Venus reporter shows increased expression in Erk2 −/− ES cells. (A) Schematic (not to scale) depicting the final Tcfcp2l1-Venus reporter construct. An 8.5 kb fragment of the mouse Tcfcp2l1 gene was cloned upstream of a splice-acceptor (SA) and GTX-IRES (IRES1), followed by a destabilized Venus protein (Venus-PEST), an ECMV-IRES (IRES2), a puromycin-resistance gene (PAC), and a polyadenylation signal (pA). (B) FACS analysis of Erk2 −/− ES cells and ERK2-rescued Erk2 −/− ES cells with the Tcfcp2l1-Venus reporter showed that Venus expression in self-renewing conditions was highest in the absence of ERK2. (C) Down-regulation of Venus expression over 48 h of differentiation followed similar kinetics in the presence or absence of ERK2. (TIF) [file pone.0060907.s004.tif]
