# Supplementary material for: ERK2 Suppresses Self-Renewal Capacity of Embryonic Stem Cells, but Is Not Required for Multi-Lineage Commitment
Source: PLoS One. 2013 Apr 16;8(4):e60907. doi: 10.1371/journal.pone.0060907 (PMC3628700; doi:10.1371/journal.pone.0060907)
Supplement: Table S1 — PCR primers for qRT-PCR analysis. Forward (F) and reverse (R) primer sequences (5′-3′) and Roche Universal Probe Library (UPL) probes used in qRT-PCR assays. (PDF) [file pone.0060907.s005.pdf]

Table S1 Primers used for qRT-PCR

Gene	Primers	UPL probe
Tbp	F: GGGGAGCTGTGATGTGAAGT	#97
	R: CCAGGAAATAATTCTGGCTCA	
Erk1	F: TGGAAGCCATGAGAGATGTTT	#94
	R: GCTCAGCTGCTGGCTTTTA	
Erk2	F: CAGAGCTCCAGAAATTATGTTGAA	#54
	R: GGGAAGATAGGCCTGTTGG	
Egr1	F: CCCTATGAGCACCTGACCAC	#22
	R: TCGTTTGGCTGGGATAACTC	
Egr2	F: CTACCCGGTGGAAGACCTC	#60
	R: AATGTTGATCATGCCATCTCC	
cFos	F: GGGACAGCCTTTCCTACTACC	#67
	R: GATCTGCGCAAAAGTCCTGT	
Dusp6	F: CTGGTGGAGAGTCGGTCCT	#66
	R: CGGCCTGGAACTTACTGAAG	
Ccnd2	F: CACTACCAGTTCCCACTCCAG	#45
	R: CTGTGCATTTACACCGACAAC	
Ccnd3	F: GGAAGATGCTGGCATACTGG	#88
	R: GGTAGCGATCCAGGTAGTTCA	
Oct4	F: GTTGGAGAAGGTGGAACCAA	#95
	R: CTCCTTCTGCAGGGCTTTC	
Nanog	F: CCTCCAGCAGATGCAAGAA	#25
	R: GCTTGCACTTCATCCTTTGG	
Acta2	F: CCAGCACCATGAAGATCAAG	#58
	R: TGGAAGGTAGACAGCGAAGC	
T	F: CAGCCCACCTACTGGCTCTA	#100
	R: GAGCCTGGGGTGATGGTA	
Gata4	F: TTCGCTGTTTCTCCCTCAAG	#60
	R: CAATGTTAACGGGTTGTGGA	
Foxa2	F: GAGCAGCAACATCACCACAG	#77
	R: CGTAGGCCTTGAGGTCCAT	

Rex1	F: CACACTCACTCTATTGAGAGAAGA	#16
	R: CAGCTCCTGCACACAGAAGA	
NeuroD3	F: CCTTCTTTGTGACTGGCTCA	#83
	R: CCCTTTTCCAAACCACACTG	
Ngn2	F: ACATCTGGAGCCGCGTAG	#69
	R: CCCAGCAGCATCAGTACCTC	
Mash1	F: TCTCCTGGGAATGGACTTTG	#74
	R: CGTTGGCGAGAAACACTAAAG	
Tbx3	F: TTGCAAAGGGTTTTCGAGAC	#51
	R: TGCAGTGTGAGCTGCTTTCT	
Klf4	F: CGGGAAGGGAGAAGACACT	#62
	R: GAGTTCCTCACGCCAACG	
Nestin	F: CTGCAGGCCACTGAAAAGT	#2
	R: TTCCAGGATCTGAGCGATCT	
Tubb3	F: GCGCATCAGCGTATACTACAA	#104
	R: TTCCAAGTCCACCAGAATGG	
Gata6	F: GGTCTCTACAGCAAGATGAATGG	#40
	R: TGGCACAGGACAGTCCAAG	
Pdgfra	F: GTCGTTGACCTGCAGTGGA	#80
	R: CCAGCATGGTGATACCTTTGT	

Forward (F) and reverse (R) primer sequences (5’-3’) and UPL probes used in qRT-PCR assays.
